# Obstructive sleep apnea and cognition: insights gleaned from bibliometric analysis

**DOI:** 10.3389/fpsyt.2023.1259251

**Published:** 2023-09-29

**Authors:** Jiajia Dong, Xiao Yu, Yuxin Wang, Honglei Zhang, Rui Guo

**Affiliations:** ^1^Department of Otorhinolaryngology Head and Neck Surgery, Air Force Medical Center, Air Force Medical University, Beijing, China; ^2^Graduate School, Hebei North University, Zhangjiakou, China; ^3^Department of Cardiovascular Medicine, Air Force Medical Center, Air Force Medical University, Beijing, China; ^4^Department of Otorhinolaryngology Head and Neck Surgery, Beijing Tiantan Hospital, Capital Medical University, Beijing, China

**Keywords:** obstructive sleep apnea, cognition, sleep, bibliometrics, CiteSpace

## Abstract

**Objective:**

Obstructive sleep apnea (OSA) is associated with cognitive impairment. However, the broad trends of the research publications on OSA and cognition are unclear. This study aimed to investigate patterns of research on the relationship between OSA and cognitive function using bibliometric analysis and to identify future research directions by analyzing research trends and emerging hotspots in the field.

**Methods:**

We searched Web of Science for relevant publications from 2003 to 2022 and conducted a bibliometric analysis of OSA and cognitive research using CiteSpace, R, and VOSviewer.

**Results:**

A total of 1995 articles met the eligibility criteria for the analysis of OSA and cognition research. There was a notable increase in publications over time, with significant contributions from the United States, particularly Harvard University, leading to substantial academic impact. Gozal D emerged as the most prolific author (59 articles) and influential researcher (3,612 citations) in this field. Hotspot analysis revealed that investigating the pathological physiology and mechanisms of OSA-associated cognitive dysfunction is a recent area of focus, while burst detection analysis identified sleep quality and mild cognitive impairment as top investigation topics. The study by Canessa N published in the American Journal of Respiratory and Critical Care Medicine received the highest number of 77 citations.

**Conclusion:**

Researchers are increasingly focusing on OSA and cognition. Currently, the majority of studies on OSA-related cognitive dysfunction are focused on correctable aspects of the condition. Future investigations into the pathology of OSA-induced cognitive impairment will facilitate more precise therapeutic interventions.

## 1. Introduction

Obstructive sleep apnea (OSA) is a prevalent and underdiagnosed condition worldwide. A recent study using the 2012 diagnostic criteria by the American Academy of Sleep Medicine found that approximately 936 million adults aged 30–69 years have mild to severe OSA, while 425 million adults in the same age range have moderate to severe OSA (1). OSA, characterized by fragmented sleep and intermittent hypoxemia, can affect brain activity and structure in middle-aged to older adults (2). Cognitive dysfunction is a common comorbidity of OSA, occurring in up to 25% of patients (3), and the general population is at increased risk of developing cognitive dysfunction (4, 5).

Although numerous studies have investigated the pathological physiology and mechanisms of OSA-associated cognitive dysfunction, there are still significant gaps in the understanding of this complex association. Neuroimaging studies have revealed brain changes in OSA patients, such as edematous changes, decreased gray and white matter density, and altered functional connectivity of the default mode network (6–9). Furthermore, longitudinal follow-up studies are needed to understand the progression of these neuroanatomical changes.

Treatment with continuous positive airway pressure (CPAP) has shown improvements in cognitive function for OSA patients. Despite the benefits of CPAP treatment in improving cognitive function in OSA patients (10), the duration of treatment and patient adherence affect the outcome (11). A trial examining the use of a mandibular advancement splint showed improvement in processing speed (12). There is limited data on the improvement of cognitive performance in patients with OSA with non-CPAP treatments. Innovative research methods and advanced technologies are essential to explore therapeutic strategies for preventing and treating cognitive impairment in OSA patients.

By employing bibliometric analysis, researchers can identify emerging trends, transformative studies, and novel technologies in their fields. This approach enables biomedical scientists to better grasp the bibliometric structure and the intellectual structure of the research field (13). Here, we conducted a bibliometric analysis and visualization of studies published from 2003 to 2022 on OSA and cognitive impairment to explore the research progress in this field and identify research direction for the future.

## 2. Materials and methods

### 2.1. Data source and search strategy

**Figure 1:**
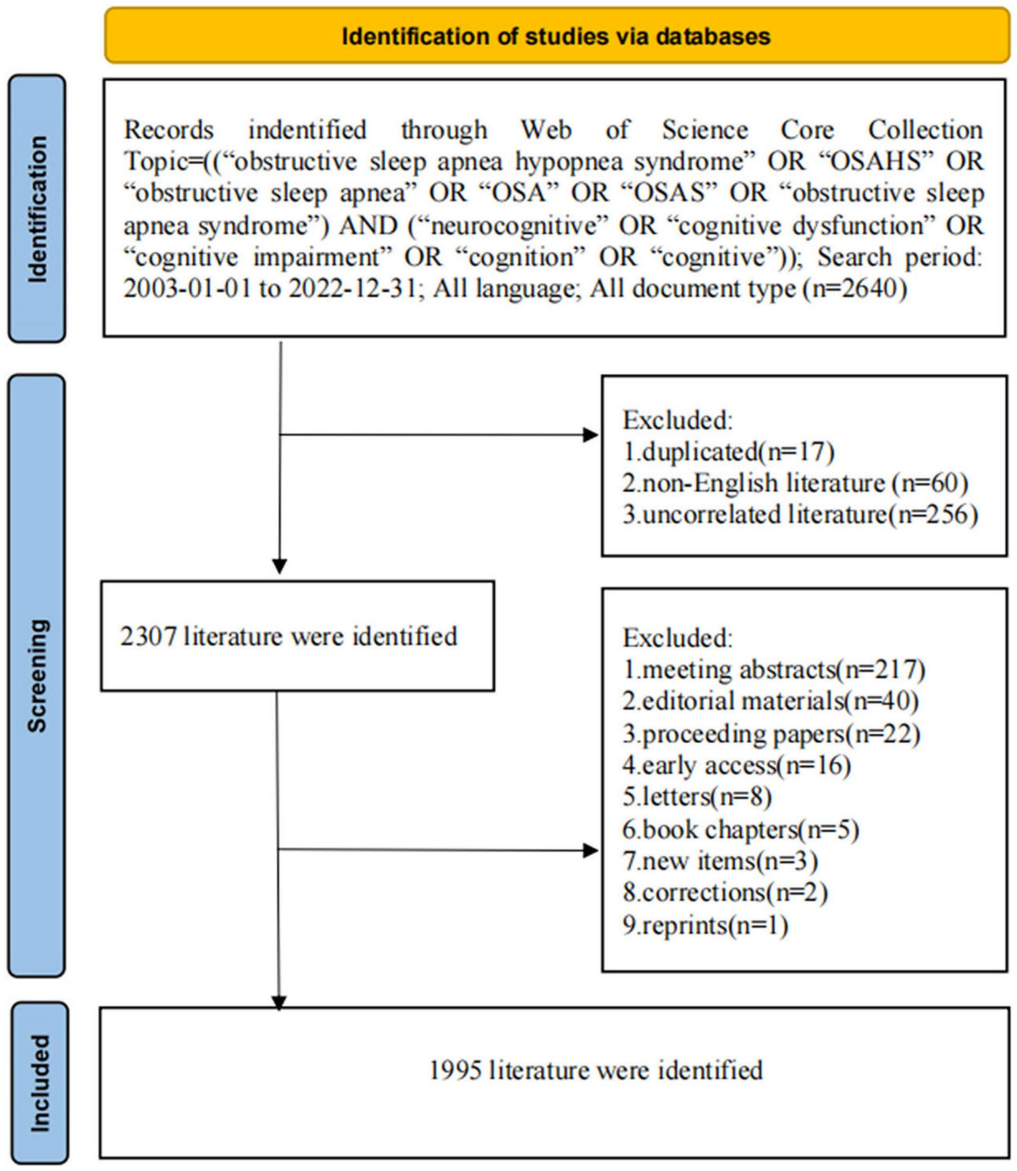
Flowchart diagram of search strategy.

The flow of the search procedure is presented in Figure 1. We attentively searched the Web of Science database for articles about OSA and cognition disease from 2003 to 2022, including original articles and review articles. The search terms were Topic = (“obstructive sleep apnea hypopnea syndrome” OR “OSAHS” OR “Obstructive Sleep Apnea” OR “OSA” OR “OSAS” OR “Obstructive Sleep Apnea Syndrome”) AND (neurocognitive” OR “cognitive dysfunction” OR “cognitive impairment” OR “Cognition” OR “cognitive”). English was the only language allowed. Two investigators (DJJ and YX) analyzed the data separately, and any conflicts were arbitrated by enlisting the assistance of a senior specialist (GR). The following information was collected: title, abstract, keywords, authors, institutions, countries, journals, references, and citations.

### 2.2. Bibliometric analysis

WoSCC included data that were converted into texts, including the basic information such as title, abstract, keywords, countries, institutions, journals, authors, and co-cited articles. The texts were imported into the analysis software.

Microsoft Office Excel 2017 software (Microsoft, Redmond, WA, USA) was used for descriptive statistical analysis of Annual Outputs and Growth Trends. Bibliometrix package of R software (version 4.2.1) was applied for bibliometric analysis and data visualization. VOSviewer software (version 1.6.19) was utilized to constitute bibliometric visualization networks of countries, institutions, authors, and keywords. Using VOSviewer co-occurrence analysis, keywords can be grouped into different “clusters” and expressed in different colors, which can forecast potential trends.

The Journal Citation Report 2022 included the impact factor, H-index, and category ranking quartiles. The H-index is defined as a useful indicator to characterize the scientific output of a journal, or researcher (14). CiteSpace software (version 6.2.R3) was developed to ascertain keywords and references with the strongest citation bursts, as well as to constitute visualization networks of co-cited references and co-cited clusters. It should be noted that different nodes on the CiteSpace software visualization network represent different analyzed individuals. The larger the nodes, the more frequently they occurred. Meanwhile, we analyzed the centrality through CiteSpace software, and the higher centrality score represented the more important node (15).

## 3. Results

### 3.1. Annual outputs and growth trends

**Figure 2:**
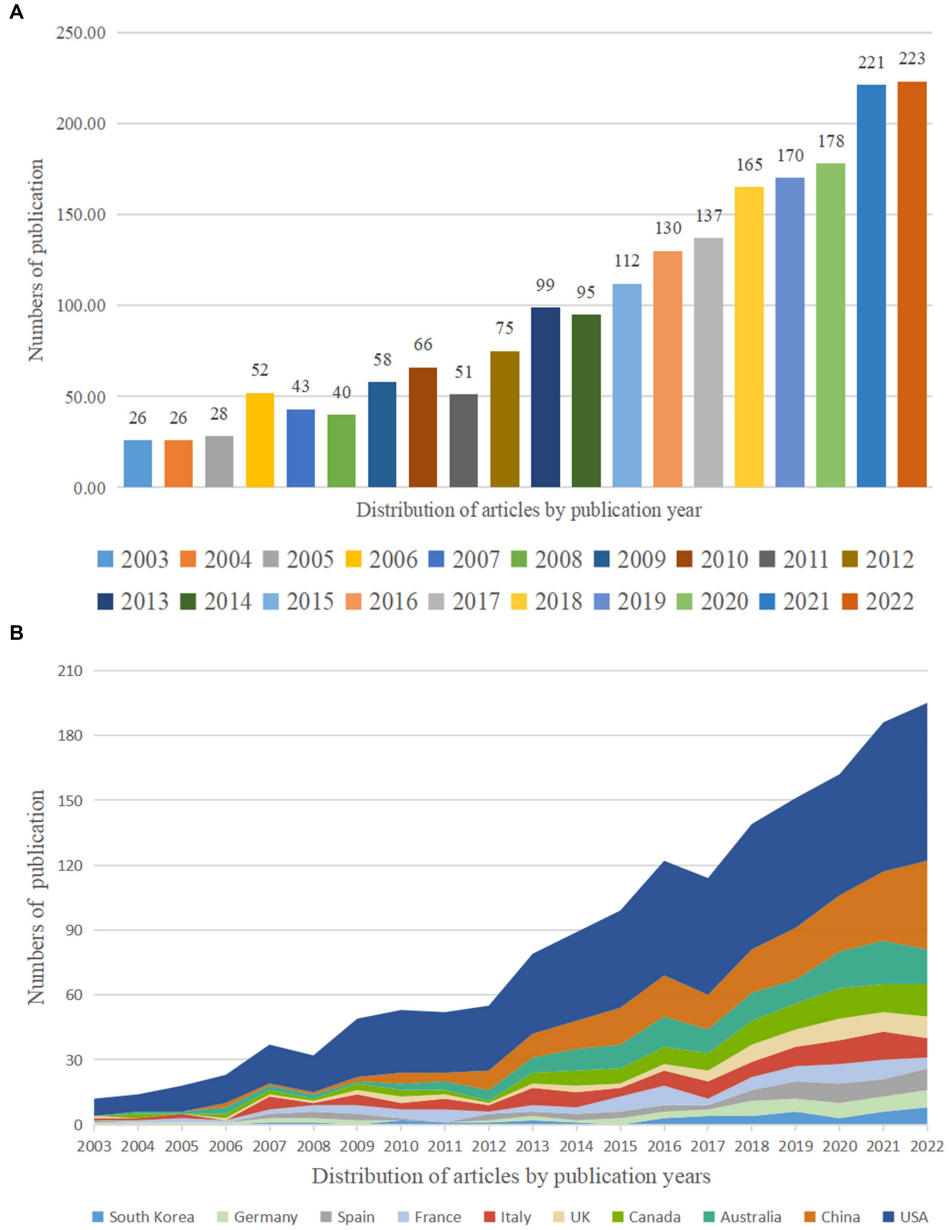
Trends in the number of publications **(A)** and the top 10 countries/regions **(B)** on OSA and cognition disease research from 2003 to 2022.

A total of 1995 articles on OSA and cognition research were published between 2003 and 2022. Research outputs related to OSA and cognition research showed an overall growing trend from 2003 to 2022 (Figure 2A). The USA published the most research outputs related to OSA and cognition research (Figure 2B). The overall number of articles published in the last 5 years, increased significantly, with 957 papers published on OSA and cognition research, accounting for 48% of the total number of papers in the past 20 years.

### 3.2. Distribution of countries/regions and institutions

**Figure 3:**
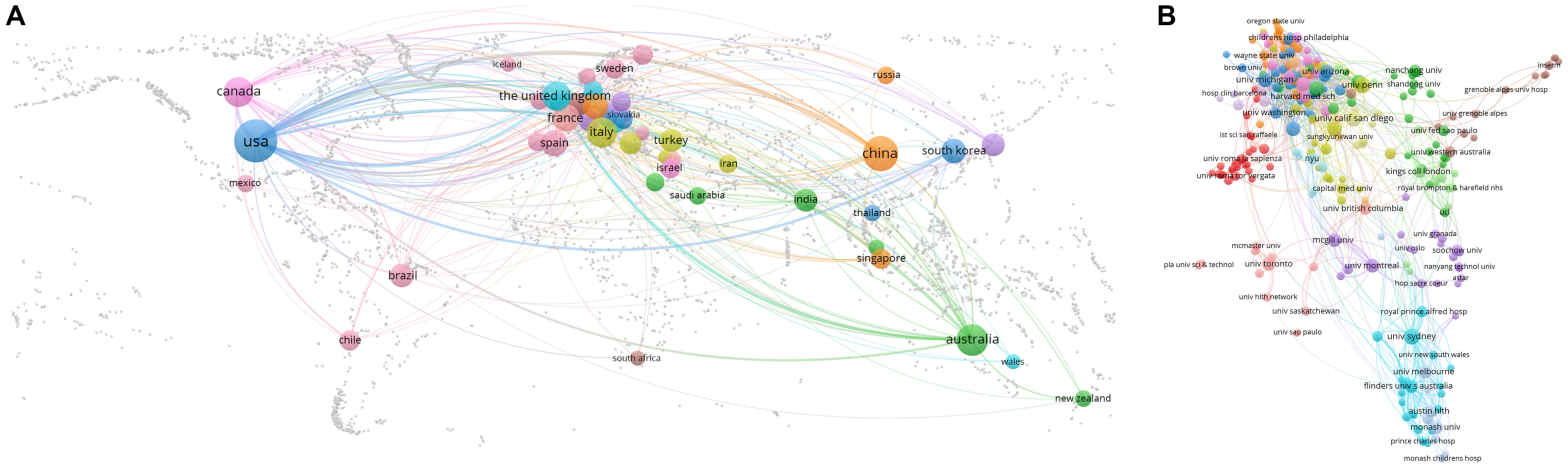
Cooperation of countries/regions **(A)** and institutions **(B)** contributed to publications on OSA and cognition disease research from 2003 to 2022.

A network of national collaborations between OSA and cognition studies was shown in Figure 3A. Table 1 showed the top ten contributing countries. Most publications came from the USA (734), followed by China (231), Australia (148), Italy (104), and Canada (92). The USA accounted for more than 40% of the total number of published studies among the top 10 countries. The centrality score is a metric for estimating the consequence of network nodes. The centrality analysis showed that the USA (0.57) was at the top of the network, followed by Germany (0.17), and Canada (0.14). Higher centrality was correlated with tighter cooperation. The low density of national collaborations in the visualization map illustrated that the research countries were relatively independent and needed further deeper cooperation. However, international collaborations between China and Japan remained rare, with only one top paper having a foreign author.

**Table 1:** Ranking of top 10 most published countries in obstructive sleep apnea and cognition research from 2003 to 2022.

Rank	Articles counts	Centrality	Country
1	734	0.57	USA
2	231	0.02	China
3	148	0.08	Australia
4	104	0.1	Italy
5	92	0.14	Canada
6	73	0.09	France
7	68	0.3	UK
8	58	0.04	Spain
9	55	0.17	Germany
10	36	0.01	South Korea

The network of institutional collaborations between studies on OSA and cognition research was shown in Figure 3B. Harvard University contributed the most articles (163) among the top 10 institutions, then the University of California System (137) and the Veterans Health Administration (128) (Table 2). 7 of the 10 most productive institutions were in the United States. The highest centrality is Harvard University (0.20), followed by the University of California System (0.14) and the University of London (0.12). Although some institutions in China contributed to a large number of papers, their number of top papers was low.

**Table 2:** Ranking of top 10 institutions for collaboration in obstructive sleep apnea and cognition research from 2003 to 2022.

Rank	Articles counts	Centrality	Institutions	Country
1	163	0.20	Harvard University	USA
2	137	0.14	University of California System	USA
3	128	0.05	Veterans Health Administration (VHA)	USA
4	69	0.08	UDICE	France
5	65	0.08	University of Pennsylvania	USA
6	63	0.07	University of Sydney	Australia
7	51	0.07	University of California San Diego	USA
8	46	0.04	Stanford University	USA
9	45	0.12	University of London	UK
10	44	0.05	University of Chicago	USA

### 3.3. Contributions of authors

**Figure 4:**
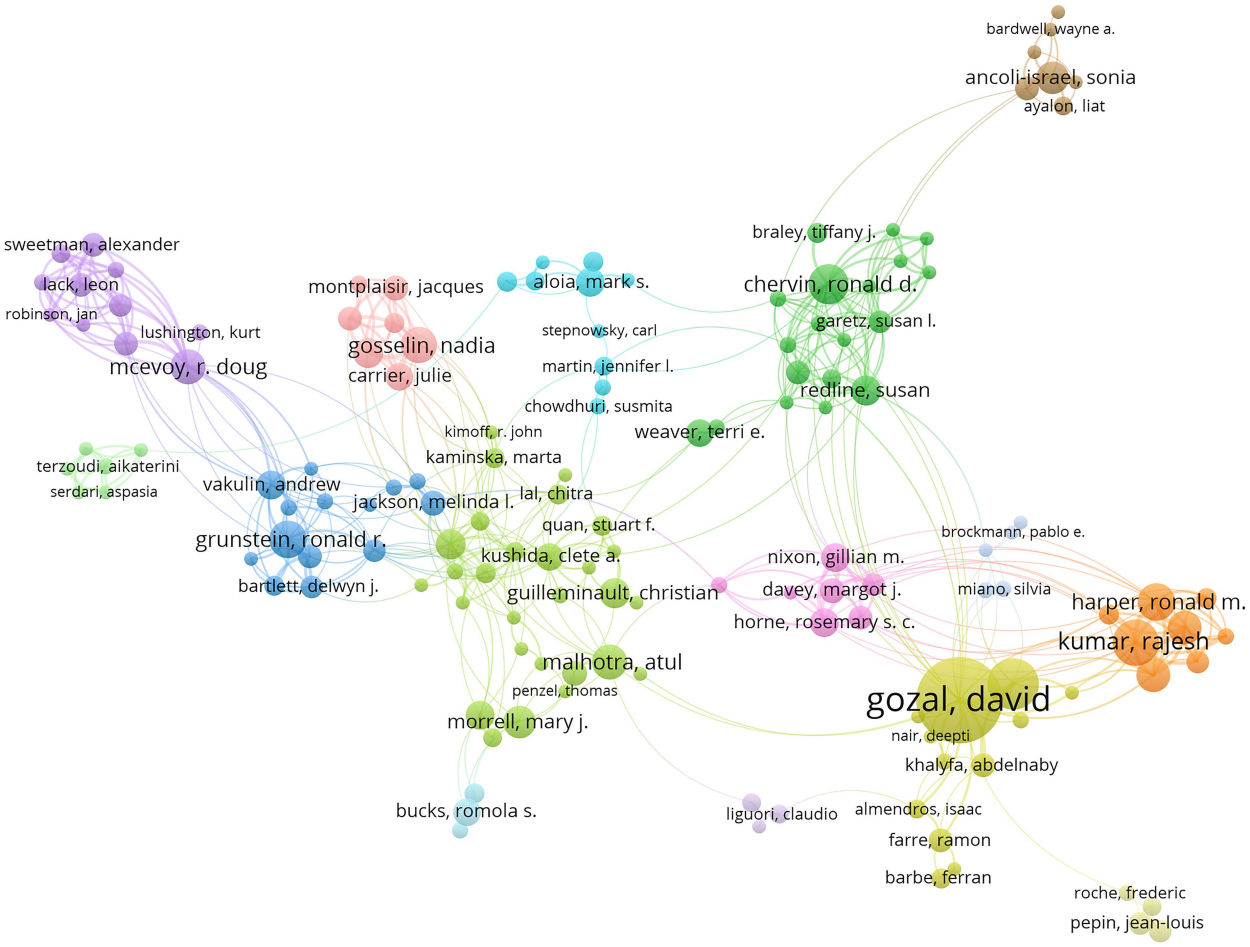
Joint mapping of high-producing authors in OSA and cognition research from 2003 to 2022.

VOSviewer was used for visualizing authors (Figure 4). A total of 9,094 authors published articles on the topic of OSA and cognition from 2003 to 2022. Of these, we found 199 authors with more than 5 publications. Each circle represented a researcher. Some overlapping authors might not be shown. Closed circles represented “active” authors who were closely associated with research partners. Table 3 showed the top 10 researchers with the most published articles. The most published author was Gozal D (59 articles, 3,612 citations), followed by Kheirandish-Gozal L (29 articles, 1,640 citations). In terms of centrality, Gozal D (0.06) was in the first place, Gosselin N (0.03) and Mcevoy RD (0.03) tied for the second echelon.

**Table 3:** Ranking of top 10 most published authors in obstructive sleep apnea and cognition research from 2003 to 2022.

Rank	Author	Articles counts	Centrality	Total citations	Average citations	H-index
1	Gozal D	59	0.06	3,612	61.22	29
2	Kheirandish-Gozal L	29	0.02	1,640	56.55	23
3	Kumar R	25	0.02	1,084	43.36	20
4	Chervin RD	20	0.02	1,259	62.95	12
5	Grunstein RR	19	0.02	751	39.53	15
6	Harper RM	19	0.01	1,009	53.11	19
7	Gosselin N	18	0.03	723	40.17	12
8	Li HJ	18	0.02	360	20.00	10
9	Mcevoy RD	17	0.03	803	47.24	14
10	Malhotra A	17	0.02	2000	117.65	14

### 3.4. Journal of analyses

Table 4 showed the characteristics of the 10 most active journals. Most of the journal publishers were published in the United States. The top 3 journals publishing OSA and cognition disease research were Sleep Medicine, SLEEP, and Journal of Clinical Sleep Medicine. Sleep Medicine published the most articles on OSA and cognition disease with the highest H-index (34). Notably, American Journal of Respiratory and Critical Care Medicine had the highest average number of citations (91.35). Journal Citation Report quartile Q1 included Sleep Medicine, Sleep, Sleep Medicine reviews, Chest, American Journal of Respiratory and Critical Care Medicine, Q2 included Journal of clinical sleep medicine, Journal of Sleep Research, and Frontiers in Neurology, and Q3 contained Sleep and Breathing, and International Journal of Pediatric Otorhinolaryngology.

**Table 4:** Ranking of top-10 journals for the number of articles published in obstructive sleep apnea and cognition research from 2003 to 2022.

Rank	Journal	Articles Count	Country	Journal citation reports (2022)	Impact factor (2022)	Total cites	Mean number of citations	H-index
1	Sleep medicine	113	Netherlands	Q1	4.8	3,617	32.01	34
2	Sleep	108	USA	Q1	5.6	3,047	28.21	30
3	Journal of clinical sleep medicine	67	USA	Q2	4.3	1970	29.40	29
4	Sleep and breathing	66	Germany	Q3	2.5	1,159	17.56	22
5	Sleep medicine reviews	46	UK	Q1	10.5	3,057	66.46	30
6	Journal of sleep research	40	UK	Q2	4.4	955	23.88	17
7	Chest	29	USA	Q1	9.6	2,170	74.83	22
8	American journal of respiratory and critical care medicine	23	USA	Q1	24.7	2,101	91.35	21
9	International journal of pediatric otorhinolaryngology	22	Netherlands	Q3	1.5	374	17.00	10
10	Frontiers in neurology	21	Switzerland	Q2	3.4	187	8.90	8

### 3.5. Keywords

#### 3.5.1. Cluster analysis of keyword co-occurrence related to research hotspots

**Figure 5:**
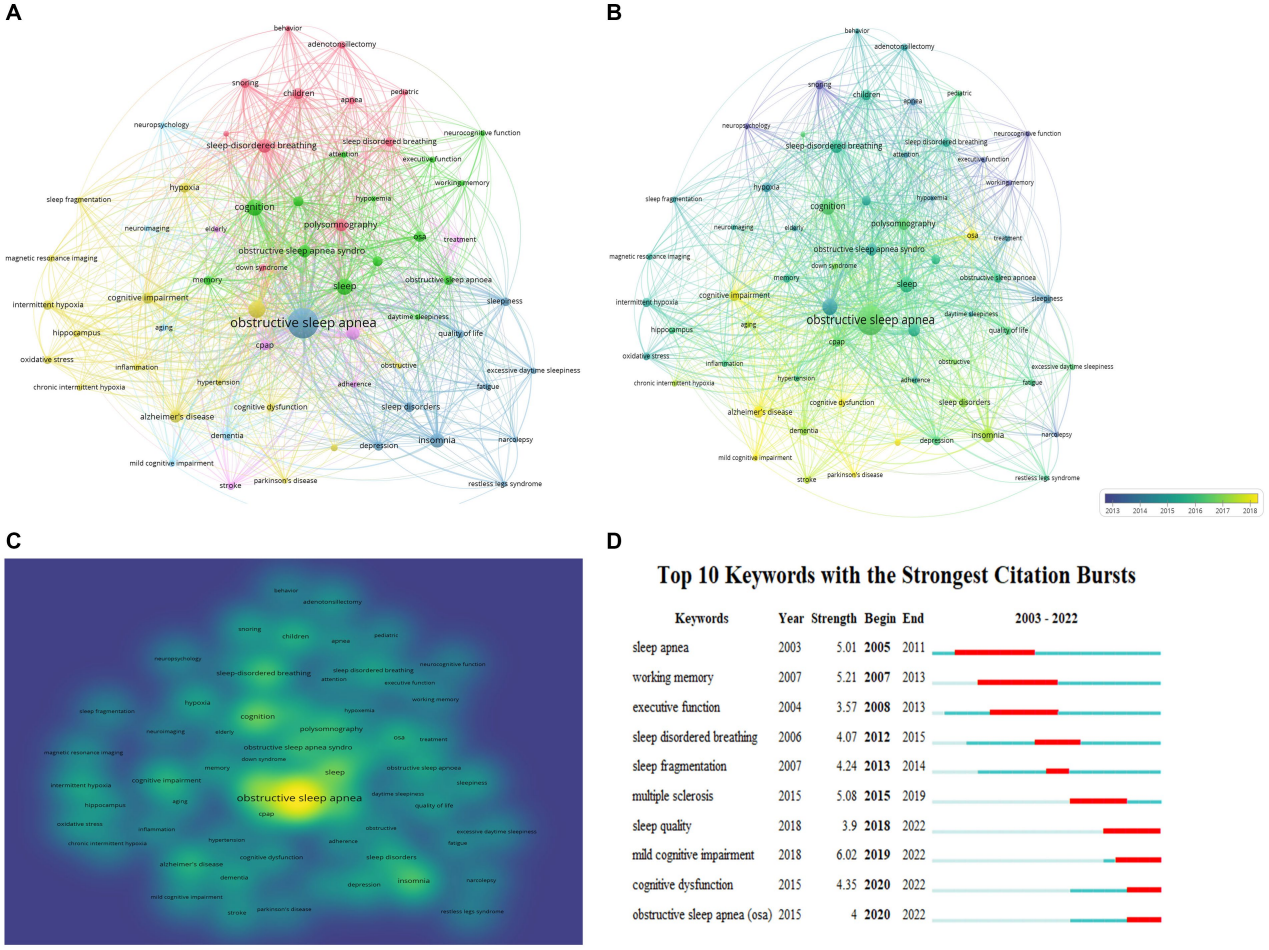
Co-occurrence analysis of global research on OSA and cognition research based on the WoSCC database from 2003 to 2022. **(A)** Mapping of keywords in the research field. **(B)** Distribution of keywords according to the chronological order of appearance. **(C)** Distribution of keywords according to the mean frequency of appearance. **(D)** Keywords with the strongest citation bursts in OSA and cognition research.

The titles and keywords of the 1995 literature included in this study were analyzed using VOSviewer software. Figure 5A showed that the map was divided into 6 clusters, containing a total of 3,555 keywords, of which 62 words were used more than 20 times. The most frequently used keywords were CPAP (103), OSA syndrome (88), Alzheimer’s disease (81), cognitive impairment (70), cognitive function (60), sleep disorders (57), and intermittent hypoxia (54). Terms with similar topics were grouped into the same category, with 6 main clusters: disorders related to cognitive impairment, clinical characteristics, mechanisms, diagnosis, treatments, and pathophysiology of OSA.

Figure 5B showed the distribution of keywords in order of their appearance. The order of the keywords was determined by the color of the labels. In the first decade, most research focused on clinical characteristics and mechanisms of OSA, and recent research trends suggest that pathological physiology and mechanisms of OSA-associated cognitive dyfunction may become a research hotspot.

Meantime, VOSviewer was utilized to measure the frequency of keywords to calculate their density, which was presented as a density map (Figure 5C). The higher the density, the warmer the color “closer to yellow.” Research hotspots in this field mostly appear in areas with high density.

#### 3.5.2. Detection of keyword bursts

By analyzing 1,995 articles retrieved from the WoSCC database, we identified keyword bursts from 2003 to 2022 (Figure 5D). A straight line represented the timeline, consisting of blue and (or) red. The red part illustrated the burst period in which length represented the start year, end year and the time span. Between 2003 and 2022, mild cognitive impairment (6.02) was top one, followed by working memory (5.21), multiple sclerosis (5.08), sleep apnea (5.01), and cognitive dysfunction (4.35). The analyses of keyword burst direction indicated that sleep quality (2018–2022), mild cognitive impairment (2019–2022), cognitive dysfunction (2020–2022), OSA (2020–2022) were potential hotspots for investigation.

### 3.6. Analysis of research hotspots

#### 3.6.1. Most co-cited papers

**Figure 6:**
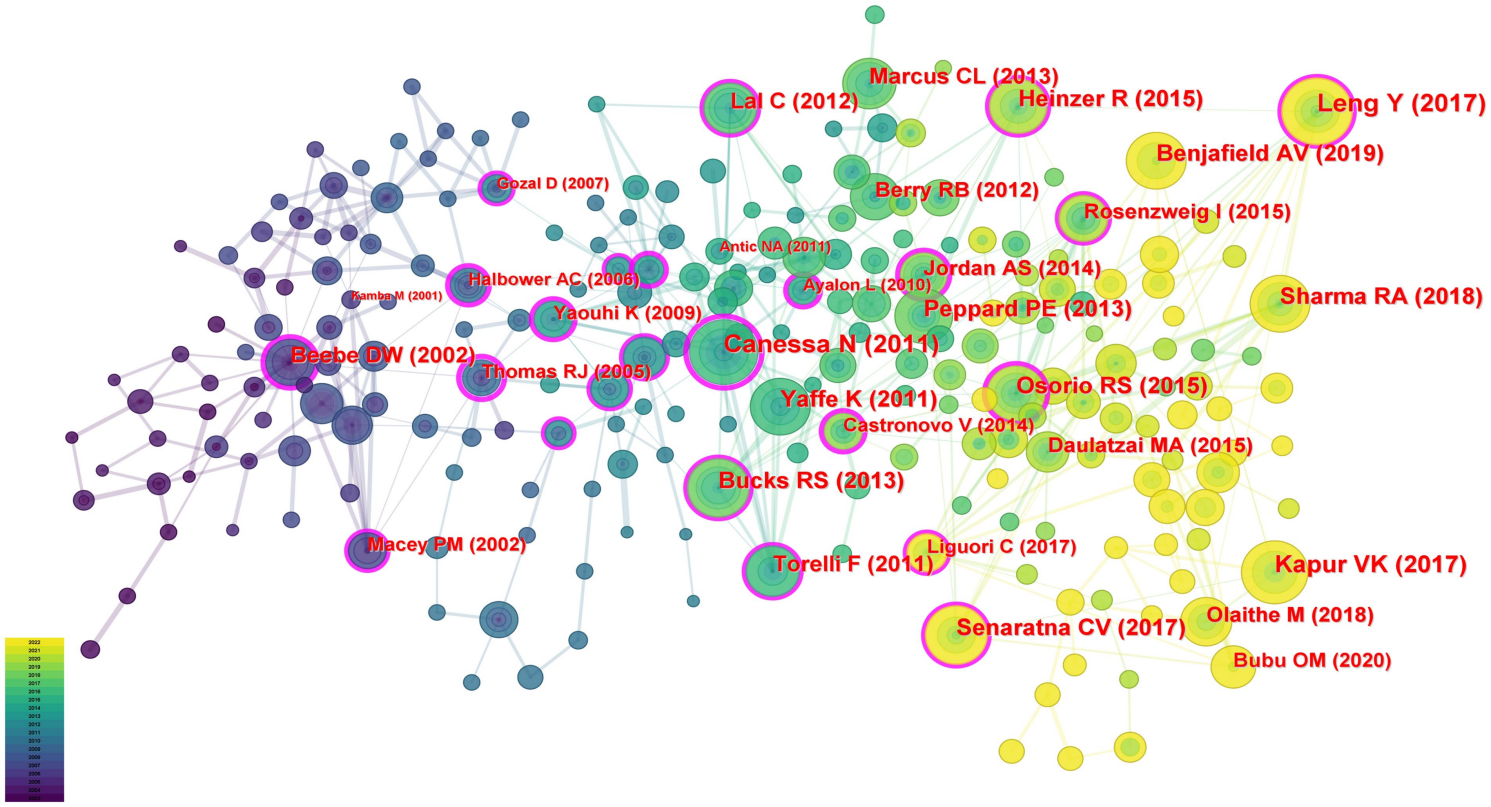
Co-cited references map on OSA and cognition research from 2003 to 2022.

In this study, 74,552 cited references including 1995 articles were analyzed for co-citation correlation, and the clustering network diagram was obtained according to the analysis results. Figure 6 showed the co-cited article visualization network with 236 nodes and 425 links. Each node indicated a cited article. The size of the node represented the total number of articles cited. The links between nodes indicated how often the same reference was cited. These nodes with a purple ring could be used to connect the growth stages of a field.

Table 5 presented the top 10 co-cited articles. The study published by Canessa N (16) in the American Journal of Respiratory and Critical Care Medicine was the most cited (77 citations), followed by the study published by Leng Y (3) in JAMA Neurology (75 citations) and the study published by Kapur VK (17) in the Journal of clinical sleep medicine (72 citations).

**Table 5:** Top 10 most co-cited references in obstructive sleep apnea and cognition research from 2003 to 2022.

Rank	Title	First author	Year	Journal	Cited frequency
1	Obstructive sleep apnea: brain structural changes and neurocognitive function before and after treatment	Canessa N	2011	American journal of respiratory and critical care medicine	77
2	Association of Sleep-Disordered Breathing With Cognitive Function and Risk of Cognitive Impairment: A Systematic Review and Meta-analysis	Leng Y	2017	JAMA neurology	75
3	Clinical Practice Guideline for Diagnostic Testing for Adult Obstructive Sleep Apnea: An American Academy of Sleep Medicine Clinical Practice Guideline	Kapur VK	2017	Journal of clinical sleep medicine	72
4	Neurocognitive function in obstructive sleep apnoea: a meta-review	Bucks RS	2013	Respirology	60
5	Prevalence of obstructive sleep apnea in the general population: A systematic review	Senaratna CV	2017	Sleep medicine reviews	57
6	Prevalence of sleep-disordered breathing in the general population: the HypnoLaus study	Heinzer R	2015	Lancet Respiratory medicine	55
7	Obstructive Sleep Apnea Severity Affects Amyloid Burden in Cognitively Normal Elderly. A Longitudinal Study	Sharma RA	2018	American journal of respiratory and critical care medicine	55
8	Sleep-disordered breathing, hypoxia, and risk of mild cognitive impairment and dementia in older women	Yaffe K	2011	JAMA	54
9	Sleep-disordered breathing advances cognitive decline in the elderly	Osorio RS	2015	Neurology	54
10	Increased prevalence of sleep-disordered breathing in adults	Peppard PE	2013	American journal of epidemiology	52

#### 3.6.2. Analyses of co-cited references

The hot point in studies of OSA and cognition research can be found by ranking the co-cited articles produced in the co-citation network.

**Figure 7:**
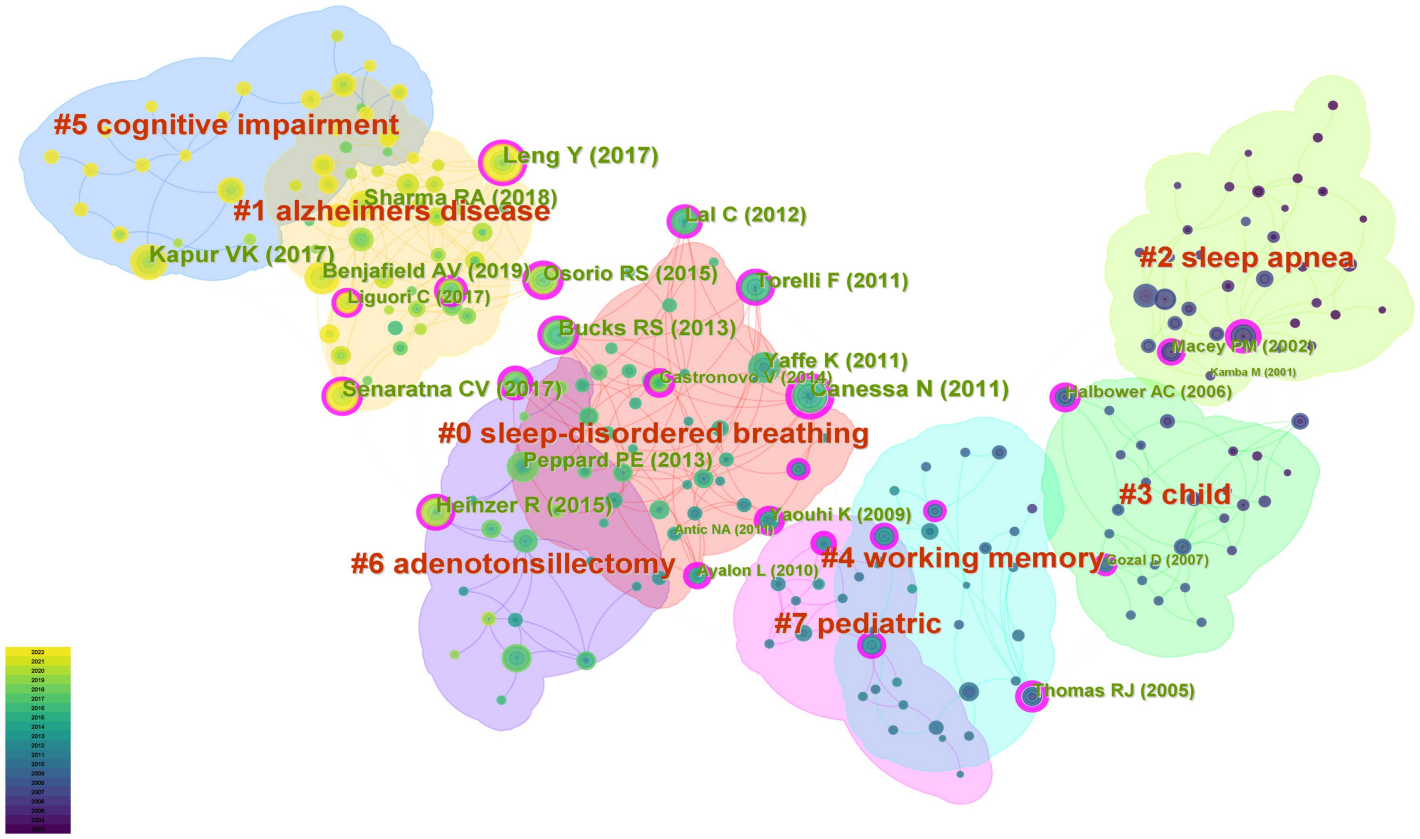
Cluster network map of co-cited references on OSA and cognition research from 2003 to 2022.

**Figure 8:**
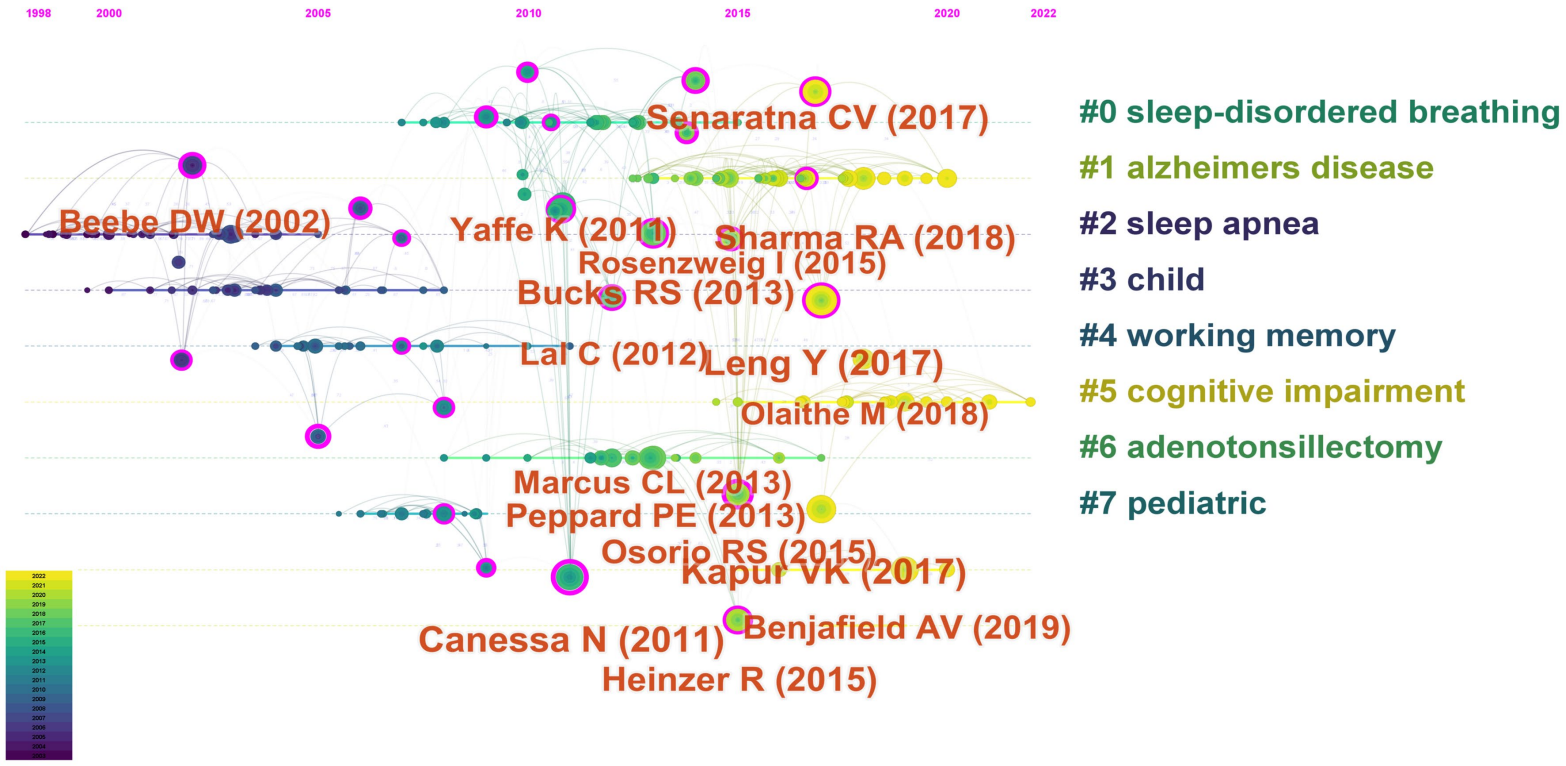
Timeline view of co-cited clusters with cluster labels.

“Sleep-Disordered Breathing,” “Alzheimer’s Disease,” “Sleep Apnea,” “Child,” “Working Memory,” “Cognitive Impairment,” “Adenotonsillectomy,” and “Pediatric” were the 8 key clusters of co-cited references (Figure 7). The timeline view of the clustering plot was shown in Figure 8, which benefited the recognition of rising research hotspots in OSA and cognition.

**Figure 9:**
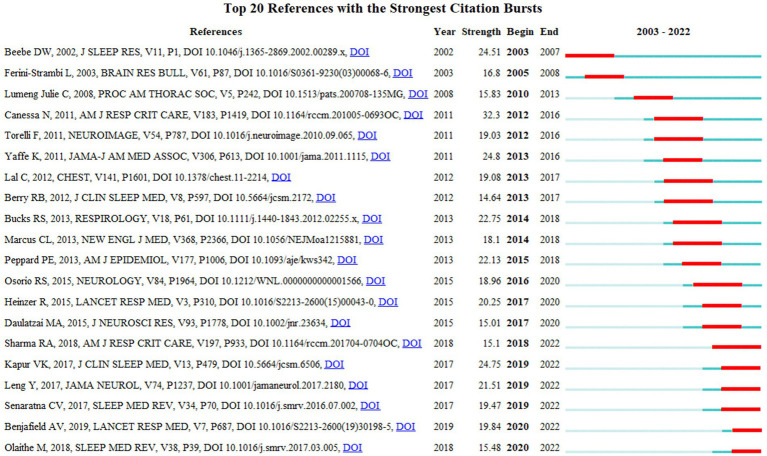
Top 20 references with the strongest citation bursts.

The top 20 most frequently cited articles were listed in Figure 9. The most frequently cited article was Obstructive sleep apnea: brain structural changes and neurocognitive function before and after treatment (16). This article with the strongest strength (32.3) was authored by Canessa N. and published in 2011. Among the top 20 cited articles, 3 articles were published in 2011 in the American Journal of Respiratory and Critical Care Medicine, Neuroimage, and JAMA. Most of the highly cited articles were published in USA journals, namely American Journal of Respiratory and Critical Care Medicine, JAMA, Journal of clinical sleep medicine, American Journal of Epidemiology, and JAMA Neurology. Most of the highly cited references were from sleep or neurocognitive publications, suggesting that cognitive was a high interest hotspot in the OSA.

## 4. Discussion

The bibliometric analysis conducted in this study revealed a significant increase in research on the link between OSA and cognitive function within clinical medicine and public health domains over the past two decades. Notably, 48% of the total articles analyzed, corresponding to 957 papers, were published in the last 5 years only. This surge highlighted the clinical relevance of OSA-related cognitive impairment and emphasized the need for continued research to advance effective therapeutic interventions. It is worth noting that this study is of great interest as it provides the first comprehensive analysis of existing literature and data, utilizes the bibliometric analysis to explore the relationship between OSA and cognitive impairment, and identifies potential future research direction.

### 4.1. Insights from analysis of OSA and cognition research

Our research indicates a growing number of studies exploring the link between OSA and cognition, highlighting the importance and advances in the field. A recent large cohort study suggested a correlation between enhanced sleep maintenance efficiency, absence of OSA, and improved cognition over a five-year period (18). It was also noted that altered OSA status affected white matter integrity and cognitive ability, exhibiting variances across age and gender (19). Specifically, in older adults over the age of 60, untreated OSA was found to impact white matter integrity and cognitive processes like visual recognition (19). However, adenotonsillectomy for treating OSA in school-age children showed limited or no impact on improving cognitive performance (20, 21). Despite the substantial advances in our understanding of the relationship between OSA and cognition, the causal role of OSA in the development of cognitive impairment remains unclear. Innovations that clarify and target the potential mechanisms of OSA-related cognitive impairment are necessary and provide a direction for future research.

Our results showed the USA had the most publications and the highest centrality scores in OSA and cognitive research, indicating that USA made important contributions to the research network and may be an influential node in collaboration and knowledge dissemination. Furthermore, Harvard University published the highest number of articles and exhibited the highest centrality score among the top 10 institutions included in the analysis, indicating the significant contribution of Harvard University to institutional collaborations in OSA and cognition research. Additionally, China, Australia, Italy, and Canada have increased their research output on OSA and cognition in recent years, indicating a positive trend toward closer international collaborations and knowledge sharing. Academic interactions among different countries and regions have become increasingly frequent and close, resulting in fruitful results in OSA and cognitive research.

Our mapping of prolific authors offers a graphic depiction of the most productive researchers in the field. From 2003 to 2022, a total of 9,094 authors contributed to publications related to OSA and cognition. Among them, Gozal D was not only the author with the highest output of articles (59 articles), but also the most cited researcher (3,612 citations) in OSA and cognition. His work has greatly increased his centrality compared to other authors, indicating that he is a key figure in this field known for high-quality research. Studying highly productive authors is beneficial for identifying emerging trends in OSA and cognition research. Moreover, the influence of academics can be evaluated based on their publication output and citation frequency, which is a measure of their scholarly ability and recognition within the academic community (22).

We also conducted an analysis of various journals. Journal analysis can be used as a reference by researchers for selecting the most appropriate journals to publish their articles. Our results showed the characteristics of the ten most active journals. Sleep Medicine, Sleep, and the Journal of Clinical Sleep Medicine ranked among the top 3 journals publishing research on OSA and cognition. In addition, we also found that the American Journal of Respiratory and Critical Care Medicine had the highest average number of citations (91.35), indicating its significant impact and recognition within the academic community. Our findings emphasize the significance of selecting publication outlets wisely and high-impact journals when seeking to disseminate research findings in the field of OSA and cognition.

### 4.2. Development status and trends in the field of OSA and cognitive research

Keyword analysis was conducted to summarize the themes and research directions within the field of OSA and cognitive research. Our results indicated that research themes related to OSA and cognitive dysfunction have become increasingly focused and interconnected over the years, suggesting a growing convergence of interest in this research area and closer relationships between different topics. By keyword analysis, OSA-related Mild cognitive impairment (MCI) has emerged as one of the most widely discussed topics in the field. MCI is frequently recognized as a pre-dementia cognitive stage of Alzheimer’s disease and is reported to develop into dementia at a rate of about 10–15% per year (23). OSA has been identified as an independent risk factor for the development of MCI in the general population. Concurrently, 37–48% of patients with OSA were found to experience MCI (24, 25). Under certain circumstances, MCI proves to be a reversible condition, with 29–55% of patients able to regain normal cognitive function (26). This highlights the importance of early detection and intervention for MCI in OSA to improve the chances of successful reversal and prevent the progression of dementia. Gagnon and colleagues recommended using MoCA to monitor MCI in OSA patients, but given the limited specificity of this test, further research is needed to identify OSA-related MCI (27). It is worth noting that CPAP can improve cognitive impairment in OSA patients, but therapeutic efficacy is limited by treatment duration and compliance (10). We also found that CPAP was the most frequently used keyword. This suggests that CPAP is a widely recognized therapeutic option for OSA and its associated cognitive dysfunction, highlighting the importance of exploring effective treatment strategies to improve prognosis.

Besides MCI, sleep quality is also an important and promising topic in the area of OSA and cognitive research. Maintaining adequate and high-quality sleep is crucial for preserving cognitive function. However, OSA-induced sleep fragmentation can reduce sleep quality (28). Poor sleep quality is a significant determinant and predictor of declining health, with consequences such as daytime sleepiness, fatigue, memory impairment, and negative mood (29). It is worth highlighting that sleep quality is modifiable and can be improved by behavioral changes, therapy, and pharmacotherapy when needed, making it an attractive target in the fields of OSA and cognitive research. Evidence demonstrates that CPAP therapy can enhance sleep quality, diminish daytime drowsiness, and boost life quality for individuals experiencing symptomatic OSA. Continued utilization of CPAP for 12 weeks improves cognitive function and sleep quality in patients with OSA (30).

Recent research trends suggest that pathological physiology and mechanisms of OSA-associated cognitive dysfunction may become a research hotspot. OSA is clinically associated with biomarkers of attention deficit disorder pathology in middle-aged and elderly patients. These include amyloid protein detected by PET imaging, Aβ and tau levels in cerebrospinal fluid and serum, neurofilament light chain in cerebrospinal fluid, and plasma cytokines (23). It is predominantly believed that cognitive deficits in OSA can be attributed to disruptions in sleep architecture and fluctuations in blood oxygen saturation. For instance, OSA-induced sleep fragmentation could potentially increase neural activity within specific regions and reduce glymphatic flow, resulting in poor clearance of amyloid and tau. Similarly, hypoxemia resulting from OSA may directly lead to neural damage (31–33). In addition, deficits in specific domains of cognition, such as attention, memory, visuospatial/structural abilities, language, psychomotor functioning, and executive functioning, were found in patients with OSA (19, 34, 35). In particular, deficits in attention, memory, executive function, psychomotor function, and language abilities observed in untreated OSA may be primarily influenced by hypoxia, whereas those related to attention and memory could be a result of sleep disturbance (35). In line with this understanding, and taking into account the detrimental effects of OSA on cognitive functioning, an adequate duration of CPAP treatment (12 months) has been demonstrated to fully reverse the white matter abnormalities in OSA patients, with corresponding improvements in impairments in memory, attention, and executive functioning (36). Thus, these results imply that cognitive deficits seen in cognitive deficits occurred in OSA may be due to damage to brain regions associated with these tasks. However, current advances are still insufficient for deeply illustrating OSA-associated cognitive impairment. More explorations are needed for deciphering the OSA-related cognitive deficits. A pivotal step would be the identification and validation of biomarkers specific to OSA-related cognitive decline. These biomarkers could possibly illuminate the complex connections between OSA and cognitive deficits, revealing new therapeutic opportunities.

Citation analysis is a useful method for identifying the most influential studies in a given research field and the academic connections among published researches (37). Citation frequency serves as a crucial indicator for evaluating the influence and quality of research papers and is commonly used to assess the impact and quality of scientific research conducted by individuals, institutions, or countries (38). The most frequently co-cited article is Obstructive sleep apnea: brain structural changes and neurocognitive function before and after treatment (16). The article focused on cognitive deficits, brain morphology changes in OSA, and the modifications after treatment. It revealed that repetitive episodes of hypoxia and sleep fragmentation associated with OSA may cause cognitive and structural impairments, which can be improved by receiving consistent and comprehensive treatment.

### 4.3. Strengths and limitations

One of the strengths of this study is the informative and instructive application of bibliometric analysis to examine the relationship between OSA and cognitive impairment. Indeed, bibliometric analysis is prevalent in both clinical practice and research. For instance, within the medical field, the utilization of bibliometric analysis allows for the tracing of significant historical origins (13). Moreover, bibliometric analysis can facilitate collaboration by identifying shared clinical practices and research interests, as well as academic affiliations, areas of expertise, and available resources (39).

A potential limitation of this study is that it only analyzed publications indexed in the Web of Science. It is important to acknowledge that different databases may yield slightly different results, potentially limiting the complete generalizability of our conclusions. Furthermore, we should note that bibliometric analysis is primarily designed to assess the scientific output and the research performance of scientific publications, rather than to provide a comprehensive overview of cognition affected by OSA. In future research, we will summarize and analyze the findings in this area to facilitate a more in-depth exploration of this research topic. In addition, our analytical framework restricts our enumeration of studies on pediatric, adult, and geriatric patients with OSA. Nevertheless, we recognize the importance of such age-specific delineation for fully comprehending cognition in OSA. In follow-up, we will address this issue by conducting targeted bibliometric assessments of different age groups.

### 4.4. Summary

In summary, our bibliometric analysis revealed that studies on cognition functioning in OSA have primarily focused on MCI and sleep quality. Our study also highlights the need for future research to investigate the pathological physiology and underlying mechanisms of OSA-related cognitive dysfunction. Overall, our findings contribute to identifying research trends and potential therapeutic targets in this field, providing guidance for future studies.

## Data availability statement

The raw data supporting the conclusions of this article will be made available by the authors, without undue reservation.

## Author contributions

JD: Methodology, Software, Writing – original draft, Writing – review & editing, Data curation, Investigation. XY: Methodology, Software, Visualization, Writing – original draft, Writing – review & editing. YW: Data curation, Methodology, Software, Writing – review & editing. HZ: Data curation, Methodology, Writing – review & editing. RG: Investigation, Project administration, Supervision, Writing – review & editing.

## Funding

The author(s) declare that no financial support was received for the research, authorship, and/or publication of this article.

## Conflict of interest

The authors declare that the research was conducted in the absence of any commercial or financial relationships that could be construed as a potential conflict of interest.

## Publisher’s note

All claims expressed in this article are solely those of the authors and do not necessarily represent those of their affiliated organizations, or those of the publisher, the editors and the reviewers. Any product that may be evaluated in this article, or claim that may be made by its manufacturer, is not guaranteed or endorsed by the publisher.
